# Anxiety genetics – findings from cross-species genome-wide approaches

**DOI:** 10.1186/2045-5380-3-9

**Published:** 2013-05-09

**Authors:** Ewa Sokolowska, Iiris Hovatta

**Affiliations:** 1 Department of Biosciences, Viikki Biocenter, University of Helsinki, Helsinki, Finland; 2 Mental Health and Substance Abuse Services, National Institute for Health and Welfare, Helsinki, Finland

**Keywords:** Anxiety disorders, Anxiety-like behavior, Mouse model, Cross-species approach, Genome-wide association study, Quantitative trait locus, Gene expression, Proteomics, Candidate gene

## Abstract

Anxiety disorders are complex diseases, which often occur in combination with major depression, alcohol use disorder, or general medical conditions. Anxiety disorders were the most common mental disorders within the EU states in 2010 with 14% prevalence. Anxiety disorders are triggered by environmental factors in genetically susceptible individuals, and therefore genetic research offers a great route to unravel molecular basis of these diseases. As anxiety is an evolutionarily conserved response, mouse models can be used to carry out genome-wide searches for specific genes in a setting that controls for the environmental factors. In this review, we discuss translational approaches that aim to bridge results from unbiased genome-wide screens using mouse models to anxiety disorders in humans. Several methods, such as quantitative trait locus mapping, gene expression profiling, and proteomics, have been used in various mouse models of anxiety to identify genes that regulate anxiety or play a role in maintaining pathological anxiety. We first discuss briefly the evolutionary background of anxiety, which justifies cross-species approaches. We then describe how several genes have been identified through genome-wide methods in mouse models and subsequently investigated in human anxiety disorder samples as candidate genes. These studies have led to the identification of completely novel biological pathways that regulate anxiety in mice and humans, and that can be further investigated as targets for therapy.

## Review

### Anxiety disorders

Anxiety and fear are normal emotional responses to threatening situations. In anxiety disorders these responses are exaggerated or prolonged and disturb daily life. Anxiety disorders, including panic disorder, obsessive-compulsive disorder (OCD), post-traumatic stress disorder (PTSD), social phobia, specific phobias, and generalized anxiety disorder (GAD), were the most common mental disorders within the EU states in 2010 with 14% prevalence [1]. Anxiety disorders are currently treated with drugs and/or cognitive behavioral therapy or other psychosocial treatments. Current pharmacotherapeutic options including benzodiazepines and selective serotonin reuptake inhibitors are not optimal due to addictive properties, development of tolerance, or poor efficacy in some patients. Therefore, new and better anxiolytics are needed, and their development requires understanding of the molecular mechanisms that regulate anxiety. Genetics offer an ideal route to the molecular background of anxiety as any identified genes can directly be linked to their function within the cell and the neural circuits.

Anxiety disorders are complex diseases caused by a combination of genetic and environmental factors. In recent years, several genes have been associated with anxiety disorders [2]. Replicated associations exist to genes belonging to various neurotransmitter or neuropeptide systems [3]. Recently, the first genome-wide association studies (GWAS) aiming to identify common variants have been published in anxiety-related personality trait neuroticism and panic disorder [4-7]. These studies support involvement of a relatively large number of small effect size common and rare variants in the predisposition to anxiety disorders, a notion shared with other psychiatric diseases, such as schizophrenia and major depression. Therefore, very large sample sizes (several thousands of individuals) will be needed to identify variants predisposing to anxiety disorders.

Anxiety is an evolutionarily conserved response and can be reliably measured in mice (Table 1). The advantage of mouse models is that the environmental factors can be controlled for, or specifically administered. In addition, brain tissue can be collected at any time point. To complement human genetic studies several groups have used mouse models of anxiety-like behavior for identification of genes and biological pathways that regulate anxiety. In general two approaches can be taken: i) candidate gene studies have mostly used transgenic models to investigate a role of a specific gene in the regulation of anxiety, and ii) genome-wide approaches do not make any prior assumptions regarding which genes contribute to the phenotype. In this review, we will concentrate on genome-wide approaches in mice, which have resulted in the identification of genes regulating anxiety. We have further restricted our focus to those genes that have subsequently been associated at some level to human anxiety disorders. Therefore, several interesting genes that may regulate anxiety but i) have been identified initially through transgenic mouse models, ii) human candidate gene or GWAS studies, or iii) have been identified in mouse models but not shown any link to human anxiety disorders, are not discussed here [8-10].

**Table 1:** Comparison of human anxiety disorders to anxiety-like behavior in mice

**Disorder**	**Human symptoms**	**Observed behavior in mice**	**Behavioral test in mice**
Generalized anxiety disorder	Excessive worry about everyday life, leading to difficulties in concentration	Decreased social interaction, impaired sustained attention	OF, L/D, Y-maze
Posttraumatic stress disorder	Repeated re-experiencing traumatic events, leading to avoidance of stimuli associated with trauma	Increased freezing response to fear conditioning, decreased fear extinction, more pronounced spontaneous recovery	Cue and contextual fear conditioning, fear extinction
Obsessive-compulsive disorder	Intrusive thoughts that produce repetitive behavior aimed at reducing anxiety	Increased marble burying and excessive grooming	Burrowing test, nest construction test
Social phobia	avoidance of social contact, emotional discomfort caused by presence of unknown people	Low social interaction	Three-chamber test of sociability, social recognition test
Panic disorder	Intense fearfulness of sudden onset, respiratory distress	Increased escape from an aggressor	Resident intruder test
Agoraphobia	Avoidance of wide-open or crowded space	Avoidance of exposed, bright areas	OF, L/D

Anxiety disorders are classified according to the Diagnostic and Statistical Manual of Mental Disorders of American Psychiatric Association (DSM-IV). *OF*, Open field test; *L/D*, Light/dark box test. Modified from [11].

### Anxiety is an evolutionarily conserved response

Why can we use the mouse to model aspects of human anxiety disorders? Neuroevolutionary studies have shown that anxiety is an adaptive response that has been conserved during evolution [12,13]. From this perspective anxiety is viewed as a behavioral state, which occurs in response to signals of danger. On the physiological level these signals initiate activation of the hypothalamus-pituitary-adrenal (HPA) axis [14] and secretion of adrenal steroids called stress hormones, which are present in almost every vertebrate cell [15]. This leads to increased heart rate, deeper breathing, vigilance, decrease in feeding, and exploration of environment [16]. The genes that code for stress hormones are highly conserved across diverse species: primates, rodents, reptiles, and amphibians [17,18].

**Figure 1:**
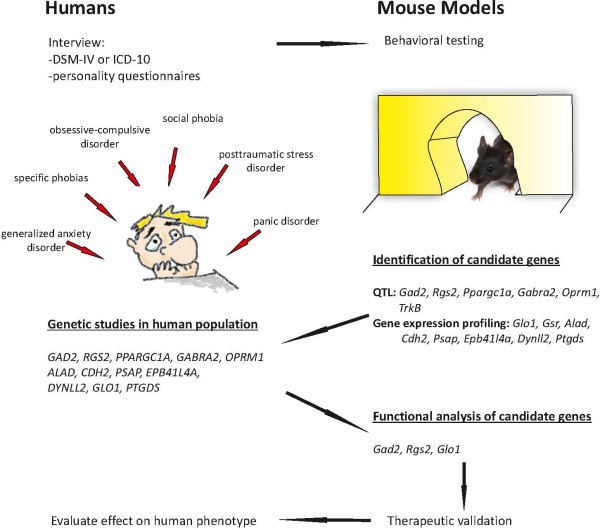
**A strategy for a cross-species mouse and human genetic approach to identify susceptibility genes for anxiety disorders.** The greatest advantages of using mouse models are the ability to reduce genetic heterogeneity and to control for the environment. The first step is to characterize aspects of a human disease in a mouse model, and to perform a search for candidate genes in a mouse model. It is then important to study these candidates in human populations to determine if they predispose to the disease under study. Mouse models are, however, needed to further characterize the function of the candidate genes, and to carry out potential drug target validation. Examples of candidate genes identified using different approaches in mice and humans are shown. Modified from [2].

Mice represent a good model system for human anxiety disorders for several reasons: i) they have a central nervous system (CNS) that is sufficiently developed to model aspects of human anxiety as compared to lower organisms, ii) hundreds of inbred strains are available, and the whole genome sequence of 17 strains has been determined [19], iii) transgenic techniques to manipulate the genome are well established, and iv) their maintenance is cost-effective. The majority of the anxiety-related behavioral tests utilize approach-avoidance behaviors that appear to mirror rodent’s behavioral response to conflict in its natural environment. Both approach behaviors, such as mate searching and foraging, and avoidance behaviors, such as escape from the predator, are evolutionarily conserved in some forms from nematodes to mammals [20]. Furthermore, the neural organization of behaviors underlying fearful, sexual, feeding, and escape motivation is relatively similar across species [21]. Disturbed balance in approach-avoidance behaviors is a symptom of autism [22], PTSD [23], and social phobia [24]. Several paradigms to test anxiety in mice, based on the approach-avoidance behavior, have been developed and pharmacologically validated with drugs that are used to treat human disease and are therefore considered appropriate models for human anxiety [25]. The most commonly used tests include the elevated plus maze, open field, light dark box, and novelty-induced hypophagia tests. In these tests mice have to choose between exploring and staying in a safe environment. However, due to cognitive differences between mouse and human, it is recognized that no animal model can mimic all aspects of human anxiety and anxiety disorders. Nevertheless, genes that regulate anxiety in mice are excellent candidate genes for anxiety disorders (Figure 1).

### Quantitative trait locus (QTL) mapping of anxiety-like behavior

QTL mapping has been used to identify genes that regulate anxiety-like behavior in rodents [26], with the idea that genes in the homologous loci in humans can then be studied as susceptibility genes for the corresponding human phenotype. Traditionally, QTL mapping has been based on genotyping F2 mice using a genome-wide marker panel and measuring the anxiety level of these animals. As a result, loci that likely contain genes affecting the phenotype can be mapped. Due to the low mapping resolution of F2 panels, other sources, such as recombinant inbred strains, heterogeneous stock mice, and outbred animals, have been used for initial and fine mapping [27-29]. It is expected that the Collaborative Cross strains, a collection of recombinant inbred mouse strains derived from eight parental strains, will be an efficient mapping resource in the future to identify both major loci and their modifiers [30]. Although initial enthusiasm for QTL mapping has been suppressed by low efficiency and resolution, and small effect size of individual variants, several anxiety-associated genes have been identified through QTL mapping [31-37]. Here we will discuss those genes that have shown some evidence for association to human anxiety disorders in subsequent studies. These include Gad2, Rgs2, Ppargc1a, Gabra2, Oprm1, and TrkB.

#### Glutamic acid decarboxylase 2 (Gad2)

One of the earliest cross-species studies investigated behavioral inhibition to the unfamiliar, a heritable temperament character that is considered a risk factor for panic and phobic anxiety [38]. Four genes were selected for genotyping in humans based on their homology to loci previously associated with anxiety or fear behavior in mice. The sample consisted of 72 behaviorally inhibited children and their family members, analyzed in a family-based association analysis. Suggestive evidence for association was found to variants in the *GAD2* gene. GAD2 is an enzyme involved in the gamma-aminobutyric acid (GABA) synthesis, and is therefore an intriguing candidate gene as abnormalities in the GABA system have been observed in anxiety disorders [39]. *GAD2* has been studied as a candidate gene for anxiety disorders in two larger subsequent studies. In the Virginia Adult Twin Study of Psychiatric and Substance Use Disorders 14 SNPs from *GAD2* were first genotyped in 188 cases with internalizing disorders (major depression, GAD, panic disorder, agoraphobia, social phobia, or neuroticism personality trait) and 188 controls. One SNP with p < 0.1 and two SNPs within the same haplotype were followed up by genotyping additional 401 cases and 351 controls but the initial finding was not replicated [40]. Another study, consisting of anxiety disorder cases (N = 268), cases with major depression (N = 541), and 541 healthy controls, tested association to 18 SNPs within *GAD2*[41]. One SNP (rs8190646) significantly associated to major depression (p = 0.00039). No association to anxiety disorders was found. To mimic the phenotype of the original study [38] association of these SNPs were also tested with behavioral inhibition in 211 anxiety disorder cases, 202 cases with major depression, and 537 controls from the same sample. Significant association to behavioral inhibition was found in the subgroups of anxiety disorder cases and healthy controls, but not to cases with major depression or all groups combined. The contradictory findings in these two larger studies may be explained by several differences, such as phenotype definition and ethnicity of the study participants. The large ongoing GWAS studies should inform whether *GAD2* variants predispose to various anxiety disorders or other psychiatric phenotypes.

#### Regulator of G-protein signaling 2 (Rgs2)

A more recent successful cross-species study concerns the genetic background of emotionality. Initially, a linkage to chromosome 1 was found by QTL mapping of DeFries mouse strains [42], and the locus was fine mapped in outbred mice [43]. This region contains the *Rgs2* gene, encoding a regulator of G protein signaling. To investigate whether *Rgs2* interacts with the functional variant, quantitative complementation method was applied, and a small-effect QTL contributing to behavioral variation in mice was identified [44]. Furthermore, knock-out mice of *Rgs2* show increased anxiety-like behavior [45]. These results indicate that Rgs2 regulate anxiety-like behavior in mice. To study the involvement of variants in *RGS2* in intermediate phenotypes of human anxiety disorders Smoller et al. studied a family based sample (119 families) of children with behavioral inhibition, 744 unrelated adults who were tested for extraversion and introversion personality traits, and 55 unrelated adults tested with the emotional face assessment during fMRI [46]. *RGS2* SNPs associated with childhood behavioral inhibition (haplotype p = 0.00003) and introversion personality trait (p = 0.007-0.05 for single SNPs, p = 0.038 for a haplotype) as well as increased activation of amygdala and insular cortex in response to watching fearful faces. In another study, four SNPs within *RGS2* showed some association to panic disorder (p = 0.02-0.05) in a sample of 173 German cases and 173 controls [47]. Also, one SNP in *RGS2* was associated to GAD in a sample of 607 adults exposed to 2004 Florida hurricane (p = 0.026) [48]. However, a recent study of 2661 individuals from the Virginia Adult Twin Study of Psychiatric and Substance Use Disorders aiming to replicate the previous findings failed to find association to three most consistently associated SNPs from these previous studies [49]. Again these discrepant results may be due to differences in the phenotype definitions or ethnic background of the samples. However, twin studies suggest that many of these phenotypes share common risk factors [50], although it is not clear how strongly they are expected to relate to specific risk alleles and their effect size.

#### Peroxisome proliferator-activated receptor gamma, coactivator 1 alpha (Ppargc1a)

Hettema et al. [51] combined data from several sources to identify and study 52 novel candidate genes for anxiety-spectrum disorders. They started with using strain distribution pattern analysis in heterogeneous stock mice that differ in anxiety-like behavior [29]. They then ranked these genes according to prior data including 1) extant linkage and knockout studies in mice, 2) a meta-analysis of human linkage scans, and 3) a preliminary human GWAS. Subsequently SNPs covering the nine top-ranked regions containing 14 genes were genotyped in a two-stage association study of subjects from the Virginia Adult Twin Study of Psychiatric and Substance Use Disorders chosen for high or low genetic loading for anxiety-spectrum phenotypes. Several SNPs within the transcriptional co-activator *PPARGC1A* associated with the anxiety phenotype. Initially PPARGC1A was discovered in the muscle cells and brown fat and characterized as a transcriptional co-activator, which stimulates mitochondrial biogenesis by increasing oxidative phosphorylation and by enhancing oxidative respiration [52]. Further studies indicated that PPARGC1A activates nuclear respiratory factor 1 (NRF1) and 2 (NRF2) [53]. These two genes are linked to oxidative stress, and involvement of oxidative stress in anxiety has been suggested by human and rodent studies, as discussed in recent reviews [54,55].

#### Gabra2, Oprm1 and TrkB in PTSD

Fear conditioning, a form of Pavlovian learning, has been used to model some aspects of PTSD. Parker et al. used an intercross of inbred mouse strains C57BL/6J x DBA/2J to identify, and an F8 advanced intercross line to fine-map, QTL associated with fear conditioning [56]. Subsequently, publicly available DNA sequence information and gene expression data were used to identify candidate genes based on the existence of non-synonymous coding polymorphisms and/or expression QTLs. Several candidate genes previously implicated in PTSD in humans were identified: gamma-aminobutyric acid receptor subunit alpha-2 (*Gabra2*), opioid receptor-mu1 (*Oprm1*), and neurotrophic tyrosine kinase (*TrkB*). GABRA2 modulates stress response [39] and SNPs within this gene have been associated with PTSD in adult patients previously exposed to child abuse [57]. OPRM1 has been linked with PTSD through different levels of μ-opioid receptor binding potential in a sample consisting of patients with PTSD (N = 16) and controls with (n = 14) or without (n = 15) combat exposure [58]. TRKB is a receptor for brain-derived neurotrophic factor (BDNF). Carriers of the Met allele of the *BDNF* Val66Met polymorphism show impaired fear extinction and disturbed fronto-amygdala activity [10]. In addition to these genes already linked to PTSD, Parker et al. found several other genes associating with fear conditioning in mice, and variants in the homologous human genes should be investigated as candidate genes for PTSD.

### Gene expression profiling in brain tissue

Functional genomics experiments represent a data-driven approach for identifying associations between a phenotype and genes or gene networks. Based on the data, specific hypotheses can be formulated and tested *in vitro* and *in vivo*. Inbred mouse strains that differ in their innate anxiety levels have been used to identify gene expression patterns that correlate with behavioral phenotypes across a number of strains [59-61]. Fernandes et al. investigated gene expression in the hippocampus of eight inbred strains, which differ in many behavioral phenotypes, and identified 200 genes showing strain differences. The strongest genetic correlation with a phenotype was found for catechol-O-methyl transferase (*Comt*), a gene previously associated with aggressive behavior [59]. A panel of eight inbred strains was used by Letwin et al. to identify strain and brain region-specific expression differences in five brain regions. They identified several glutamatergic signaling pathway-related genes correlating with anxiety-like behavior [61]. We investigated gene expression differences in seven brain regions of six inbred mouse strains that differ in their innate anxiety levels [60]. We correlated gene expression patterns from seven brain regions, known to regulate some aspects of anxiety, with behavioral anxiety-measures and identified genes with an expression pattern that correlates with anxiety-like behavior. We then functionally verified by lentivirus-mediated gene transfer (overexpression and silencing by RNAi) that two genes, glyoxalase 1 (*Glo1*) and glutathione reductase (*Gsr*) regulate anxiety in mice [60]. Since *Glo1* has been identified by several studies using various approaches, it is discussed further in the next section. The challenge with the translation of the gene expression findings to human anxiety disorders is the poor availability of good quality post mortem brain samples. Another approach is to test if DNA variants in the homologous human genes confer predisposition to anxiety disorders, but since a large number of the gene expression changes are expected to be reactive rather than causal, this approach may work better on a pathway than single gene level.

As a translational step we tested whether genetic variants in 13 genes shown to be differentially expressed between anxious and non-anxious mouse strains predispose humans to anxiety disorders. We carried out a genetic association analysis in a Finnish population-based Health 2000 Cohort consisting of 321 cases and 653 matched controls. Variants in six genes (*CDH2*, *ALAD*, *PSAP*, *EPB41L4A*, *DYNLL2*, and *PTGDS*) showed some evidence (p < 0.01) for association to anxiety disorders [62]. Interestingly, *Cdh2* was recently shown to confer susceptibility to compulsive behavior in dogs [63].

#### Glo1 has been identified through various approaches

*Glo1* was one of the genes identified through gene expression profiling in inbred strains having a higher expression level in anxious strains [60]. In the same study, its overexpression in the cingulate cortex by lentivirus-mediated gene transfer resulted in increased anxiety-like behavior, while inhibition by overexpression of an shRNA decreased anxiety-like behavior. *Glo1* was independently identified through a genome-wide search for copy number variants (CNVs) in inbred strains [64]. It was shown that the difference in *Glo1* expression between inbred mouse strains is due to a CNV, the presence of which correlates positively with anxiety-like behavior. To show a causal relationship between the CNV and anxiety-like behavior Distler et al. generated BAC transgenic mice expressing different copy numbers of *Glo1*[65]. The mice with several copies have increased anxiety-like behavior, as expected. GLO1 is a detoxification enzyme, which together with glyoxalase 2 converts cytotoxic methylglyoxal (MG) to non-toxic form [66,67]. When exploring the molecular mechanism of GLO1 underlying anxiety behavior Distler et al. found that overexpression of *Glo1* reduces MG level in the brain. Moreover, they showed that MG is an agonist of GABAA receptors, and therefore reduced levels of MG decrease GABAA receptor activation [65]. This finding conforms well to the known involvement of GABAA receptors in the regulation of anxiety. Interestingly, two proteomics studies have also linked GLO1 with anxiety-like behavior. According to these studies GLO1 is down-regulated in the brain of two separate mouse strains selectively bred for high anxiety behavior compared to their respective low-anxiety strains [68,69], a finding contradictory to the findings in the inbred strains. This surprising difference is likely due to other alleles contributing to the anxiety phenotype in these models and other factors related to the selective breeding of the strains, including differences in initial allelic frequencies, linked alleles, and drift before or during inbreeding [70]. More detailed discussion on the role of GLO1 in behavioral phenotypes is found in an excellent recent review [70].

The role of GLO1 in mental disorders has been studied in humans. Patients with major depression or bipolar disorder show reduced *GLO1* expression when in depressive state, but not during remission [71]. However, cholecystokinin-tetrapeptide (CCK-4), which is used to induce panic attacks, did not have an effect on *GLO1* mRNA levels in peripheral blood cells of 23 healthy volunteers [72]. In schizophrenia patients, rare genetic variants in *GLO1* have been associated with decreased enzyme activity and increased carbonyl stress [73]. Genetic association studies have been carried out in anxiety disorders. A common Ala111Glu substitution in *GLO1*, responsible for conformational change and decreased enzymatic activity, was investigated in 162 panic disorder patients and 288 matched controls from the Italian population [74]. Although there was no evidence of association to the overall diagnosis, some evidence was found for association with panic disorder without agoraphobia (N = 61 patients, p = 0.015). Similarly, Donner et al. failed to find strong evidence for association with this SNP and anxiety disorders in the Finnish population (p = 0.021) [62]. This functional SNP therefore does not seem to play a major role in the predisposition to anxiety disorders. Larger genetic studies are needed to find out whether other common or rare variants within *GLO1* are involved in the etiology of anxiety disorders.

### Proteomic studies in mouse models

Altogether three proteomic studies have been carried out in bidirectionally bred mouse strains for high or low levels of anxiety. In the HAB/LAB mouse model several proteins have been identified, including GLO1, discussed already in detail above [69], and another interesting enzyme, enolase-phosphatase [75]. In a different bidirectional mouse model of anxiety-like behavior Szego et al. identified alterations in serotonin receptor-associated proteins [69]. Recent proteomic analysis of rat hippocampus after psychosocial stress revealed 21 differently expressed proteins. They were involved in various cellular functions, including signal transduction, synaptic plasticity, cytoskeleton remodeling and energy metabolism [76].

Since the proteomics-based methods are developing with fast pace, it is expected that they will in the near future reveal biomarker panels to be used in biological diagnostics of psychiatric disorders, in addition to shedding light to the neurobiological mechanisms regulating anxiety.

## Conclusions

Because of their high prevalence, anxiety disorders impose high social and economic burden. Integration of data from several approaches is needed to understand the molecular mechanisms that regulate anxiety, and to develop novel pharmacological treatments. Genome-wide approaches to identify regulators of anxiety-like behavior in animal models will greatly complement the ongoing GWAS efforts in human anxiety disorders. There are two major advantages in using mouse models compared to human patient samples. Since environmental factors can be controlled for, or specifically administered in animal models, the power to detect small genetic effects is likely better in animal models compared to human cohorts. Stress, especially in childhood, is a well-established risk factor for anxiety disorders, and several mouse models for childhood stress have been recently developed. These should be investigated in several inbred genetic backgrounds, to identify gene-environment interactions in controlled circumstances. Another benefit of using animal models is the ability to harvest brain tissue at any time point. This allows taking advantage of unbiased genome-wide and proteome-wide identification of genes that regulate anxiety. With mRNA-seq and small RNA-seq it is now possible to identify all expressed genes from a given tissue, at different time points. Bioinformatic integration of this information can then be used to identify dynamic gene regulatory networks, instead of single genes. Optogenetic manipulation of specific cell types, combined with behavioral and gene expression analysis will help to detect yet more specific circuits underlying anxiety behavior. This approach will require development of better methods to dissect specific cell types and to carry out RNA-seq from very small amounts of RNA.

Results from the animal models should be used to formulate and test specific hypotheses in humans, using genetic and imaging approaches. The progress of the translation has been hindered by the relatively small size of well-characterized anxiety disorder cohorts, as can be seen with examples given above. Also, anxiety disorders as a group are phenotypically heterogeneous and it is not expected that all genetic findings replicate across all phenotypes. Integration of results from human genetic and imaging approaches with mouse genetic and functional studies will be essential to understand the neurobiological basis of anxiety disorders, a prerequisite for targeted therapies.

## Abbreviations

ALAD: δ-Aminolevulinate dehydratase; BAC: Bacterial artificial chromosome; BDNF: Brain-derived neurotrophic factor; fMRI: Functional magnetic resonance imaging; CCK-4: Cholecystokinin-tetrapeptide; CDH2: Cadherin-2; CNS: Central nervous system; CNV: Copy number variant; Comt: Catechol-O-methyl transferase; DYNLL2: Dynein light chain 2; EPB41L4A: Erythrocyte membrane protein band 4.1 like 4A; GABA: Gamma-aminobutyric acid; GABRA2: Gamma-aminobutyric acid receptor subunit alpha-2; GAD: Generalized anxiety disorder; GAD2: Glutamic acid decarboxylase 2; Glo1: Glyoxalase 1; Gsr: Glutathione reductase; GWAS: Genome-wide association study; HAB/LAB: High anxiety-like behavior/low anxiety-like behavior; HPA: Hypothalamic-pituitary-adrenal axis; MG: Methylglyoxal; NRF: Nuclear respiratory factor; OCD: Obsessive-compulsive disorder; Oprm1: Opioid receptor, mu 1; PPARGC1A: Peroxisome proliferator-activated receptor gamma coactivator 1-alpha; PSAP: Prosaposin; PTGDS: Prostaglandin D2 synthase; PTSD: Posttraumatic stress disorders; QTL: Quantitative trait locus; Rgs2: Regulator of G-protein signaling 2; SNP: Single nucleotide polymorphism; TrkB: Neurotrophic tyrosine kinase.

## Competing interests

The authors declare that they have no competing interest.

## Authors’ contributions

ES and IH contributed equally to this manuscript. Both authors read and approved the final manuscript.

## References

[B1] Wittchen HUJF Rehm J Gustavsson A Svensson M Jönsson B Olesen J Allgulander C Alonso J Faravelli C Fratiglioni L Jennum P Lieb R Maercker A van Os J Preisig M Salvador-Carulla L Simon R Steinhausen H The size and burden of mental disorders and other disorders of the brain in Europe 2010 Eur Neuropsychopharmacol 2010 21 655 679 21896369 10.1016/j.euroneuro.2011.07.018

[B2] Hovatta I Barlow C Molecular genetics of anxiety in mice and men Ann Med 2008 40 92 109 10.1080/07853890701747096 18293140

[B3] Arnold PD Zai G Richter MA Genetics of anxiety disorders Curr Psychiatry Rep 2004 6 243 254 10.1007/s11920-004-0073-1 15260939

[B4] Calboli FC Tozzi F Galwey NW Antoniades A Mooser V Preisig M Vollenweider P Waterworth D Waeber G Johnson MR A genome-wide association study of neuroticism in a population-based sample PLoS One 2010 5 e11504 10.1371/journal.pone.0011504 20634892 PMC2901337

[B5] Otowa T Yoshida E Sugaya N Yasuda S Nishimura Y Inoue K Tochigi M Umekage T Miyagawa T Nishida N Genome-wide association study of panic disorder in the Japanese population J Hum Genet 2009 54 122 126 10.1038/jhg.2008.17 19165232

[B6] Shifman S Bhomra A Smiley S Wray NR James MR Martin NG Hettema JM An SS Neale MC van den Oord EJ A whole genome association study of neuroticism using DNA pooling Mol Psychiatry 2008 13 302 312 10.1038/sj.mp.4002048 17667963 PMC4004964

[B7] Terracciano A Sanna S Uda M Deiana B Usala G Busonero F Maschio A Scally M Patriciu N Chen WM Genome-wide association scan for five major dimensions of personality Mol Psychiatry 2010 15 647 656 10.1038/mp.2008.113 18957941 PMC2874623

[B8] Chen ZY Jing D Bath KG Ieraci A Khan T Siao CJ Herrera DG Toth M Yang C McEwen BS Genetic variant BDNF (Val66Met) polymorphism alters anxiety-related behavior Science 2006 314 140 143 10.1126/science.1129663 17023662 PMC1880880

[B9] Erhardt A Czibere L Roeske D Lucae S Unschuld PG Ripke S Specht M Kohli MA Kloiber S Ising M TMEM132D, a new candidate for anxiety phenotypes: evidence from human and mouse studies Mol Psychiatry 2011 16 647 663 10.1038/mp.2010.41 20368705

[B10] Soliman F Glatt CE Bath KG Levita L Jones RM Pattwell SS Jing D Tottenham N Amso D Somerville LH A genetic variant BDNF polymorphism alters extinction learning in both mouse and human Science 2010 327 863 866 10.1126/science.1181886 20075215 PMC2829261

[B11] Cryan JF Holmes A The ascent of mouse: advances in modelling human depression and anxiety Nat Rev Drug Discov 2005 4 775 790 10.1038/nrd1825 16138108

[B12] Nesse R Emotional disorders in evolutionary perspective Br J Med Psychol 1998 71 397 415 10.1111/j.2044-8341.1998.tb01000.x 9875953

[B13] Stein DJ Bouwer C A neuro-evolutionary approach to the anxiety disorders J Anxiety Disord 1997 11 409 429 10.1016/S0887-6185(97)00019-4 9276785

[B14] Boyce WT Ellis BJ Biological sensitivity to context: I. An evolutionary-developmental theory of the origins and functions of stress reactivity Dev Psychopathol 2005 17 271 301 16761546 10.1017/s0954579405050145

[B15] Korte SM Corticosteroids in relation to fear, anxiety and psychopathology Neurosci Biobehav Rev 2001 25 117 142 10.1016/S0149-7634(01)00002-1 11323078

[B16] Cannon WB Bodily changes in pain, hunger, fear and rage: Researches into the function of emotional excitement 1915/1929 New York: Harper & Row

[B17] Lovejoy DA Balment RJ Evolution and physiology of the corticotropin-releasing factor (CRF) family of neuropeptides in vertebrates Gen Comp Endocrinol 1999 115 1 22 10.1006/gcen.1999.7298 10375459

[B18] Lovejoy DA Jahan S Phylogeny of the corticotropin-releasing factor family of peptides in the metazoa Gen Comp Endocrinol 2006 146 1 8 10.1016/j.ygcen.2005.11.019 16472809

[B19] Keane TM Goodstadt L Danecek P White MA Wong K Yalcin B Heger A Agam A Slater G Goodson M Mouse genomic variation and its effect on phenotypes and gene regulation Nature 2011 477 289 294 10.1038/nature10413 21921910 PMC3276836

[B20] O’Connell LA Hofmann HA Genes, hormones, and circuits: an integrative approach to study the evolution of social behavior Front Neuroendocrinol 2011 32 320 335 10.1016/j.yfrne.2010.12.004 21163292

[B21] Elliot AJ Covington MV Approach and avoidance motivation Educ Psychol Rev 2001 13 73 92 10.1023/A:1009009018235

[B22] Lombardo MV Ashwin E Auyeung B Chakrabarti B Lai MC Taylor K Hackett G Bullmore ET Baron-Cohen S Fetal programming effects of testosterone on the reward system and behavioral approach tendencies in humans Biol Psychiatry 2012 72 839 847 10.1016/j.biopsych.2012.05.027 22763187 PMC3485553

[B23] Stein MB Paulus MP Imbalance of approach and avoidance: the yin and yang of anxiety disorders Biol Psychiatry 2009 66 1072 1074 10.1016/j.biopsych.2009.09.023 19944792 PMC2825567

[B24] Dell’Osso L Rucci P Ducci F Ciapparelli A Vivarelli L Carlini M Ramacciotti C Cassano GB Social anxiety spectrum Eur Arch Psychiatry Clin Neurosci 2003 253 286 291 10.1007/s00406-003-0442-5 14714117

[B25] Gordon JA Hen R Genetic approaches to the study of anxiety Annu Rev Neurosci 2004 27 193 222 10.1146/annurev.neuro.27.070203.144212 15217331

[B26] Ramos A Moisan MP Chaouloff F Mormede C Mormede P Identification of female-specific QTLs affecting an emotionality-related behavior in rats Mol Psychiatry 1999 4 453 462 10.1038/sj.mp.4000546 10523818

[B27] Mott R Flint J Simultaneous detection and fine mapping of quantitative trait loci in mice using heterogeneous stocks Genetics 2002 160 1609 1618 11973314 10.1093/genetics/160.4.1609 PMC1462050

[B28] Yalcin B Flint J Mott R Using progenitor strain information to identify quantitative trait nucleotides in outbred mice Genetics 2005 171 673 681 10.1534/genetics.104.028902 16085706 PMC1456780

[B29] Valdar W Solberg LC Gauguier D Burnett S Klenerman P Cookson OW Taylor MS Rawlins JNP Mott R Flint J Genome-wide genetic association of complex traits in heterogeneous stock mice Nat Genet 2006 38 879 887 10.1038/ng1840 16832355

[B30] Welsh CE Miller DR Manly KF Wang J McMillan L Morahan G Mott R Iraqi FA Threadgill DW de Villena FP Status and access to the collaborative cross population Mamm Genome 2012 23 706 712 10.1007/s00335-012-9410-6 22847377 PMC3463789

[B31] Turri MG DeFries JC Henderson ND Flint J Multivariate analysis of quantitative trait loci influencing variation in anxiety-related behavior in laboratory mice Mamm Genome 2004 15 69 76 10.1007/s00335-003-3032-y 15058378

[B32] Turri MG Datta SR DeFries J Henderson ND Flint J QTL analysis identifies multiple behavioral dimensions in ethological tests of anxiety in laboratory mice Curr Biol 2001 11 725 734 11378382 10.1016/s0960-9822(01)00206-8

[B33] Singer JB Hill AE Nadeau JH Lander ES Mapping quantitative trait loci for anxiety in chromosome substitution strains of mice Genetics 2005 169 855 862 10.1534/genetics.104.031492 15371360 PMC1449086

[B34] Ponder CA Kliethermes CL Drew MR Muller J Das K Risbrough VB Crabbe JC Gilliam TC Palmer AA Selection for contextual fear conditioning affects anxiety-like behaviors and gene expression Genes Brain Behav 2007 6 736 749 10.1111/j.1601-183X.2007.00306.x 17309658

[B35] Philip VM Duvvuru S Gomero B Ansah TA Blaha CD Cook MN Hamre KM Lariviere WR Matthews DB Mittleman G High-throughput behavioral phenotyping in the expanded panel of BXD recombinant inbred strains Genes Brain Behav 2010 9 129 159 10.1111/j.1601-183X.2009.00540.x 19958391 PMC2855868

[B36] Goodson M Rust MB Witke W Bannerman D Mott R Ponting CP Flint J Cofilin-1: a modulator of anxiety in mice PLoS Genet 2012 8 1 8 10.1371/journal.pgen.1002970 PMC3464202 23055942

[B37] Eisener-Dorman AF Grabowski-Boase L Steffy BM Wiltshire T Tarantino LM Quantitative trait locus and haplotype mapping in closely related inbred strains identifies a locus for open field behavior Mamm Genome 2010 21 231 246 10.1007/s00335-010-9260-z 20473506

[B38] Smoller JW Rosenbaum JF Biederman J Susswein LS Kennedy J Kagan J Snidman N Laird N Tsuang MT Faraone SV Genetic association analysis of behavioral inhibition using candidate loci from mouse models Am J Med Genet 2001 105 226 235 10.1002/ajmg.1328 11353440

[B39] Lydiard RB The role of GABA in anxiety disorders J Clin Psychiatry 2003 64 Suppl 3 21 27 12662130

[B40] Hettema JM An SS Neale MC Bukszar J van den Oord EJ Kendler KS Chen X Association between glutamic acid decarboxylase genes and anxiety disorders, major depression, and neuroticism Mol Psychiatry 2006 11 752 762 10.1038/sj.mp.4001845 16718280

[B41] Unschuld PG Ising M Specht M Erhardt A Ripke S Heck A Kloiber S Straub V Brueckl T Muller-Myhsok B Polymorphisms in the GAD2 gene-region are associated with susceptibility for unipolar depression and with a risk factor for anxiety disorders Am J Med Genet B Neuropsychiatr Genet 2009 150B 1100 1109 10.1002/ajmg.b.30938 19229853

[B42] Flint J Valdar W Shifman S Mott R Strategies for mapping and cloning quantitative trait genes in rodents Nat Rev Genet 2005 6 271 286 15803197 10.1038/nrg1576

[B43] Talbot CJ Nicod A Cherny SS Fulker DW Collins AC Flint J High-resolution mapping of quantitative trait loci in outbred mice Nat Genet 1999 21 305 308 10.1038/6825 10080185

[B44] Yalcin B Willis-Owen SA Fullerton J Meesaq A Deacon RM Rawlins JN Copley RR Morris AP Flint J Mott R Genetic dissection of a behavioral quantitative trait locus shows that Rgs2 modulates anxiety in mice Nat Genet 2004 36 1197 1202 10.1038/ng1450 15489855

[B45] Oliveira-Dos-Santos AJ Matsumoto G Snow BE Bai D Houston FP Whishaw IQ Mariathasan S Sasaki T Wakeham A Ohashi PS Regulation of T cell activation, anxiety, and male aggression by RGS2 Proc Natl Acad Sci U S A 2000 97 12272 12277 10.1073/pnas.220414397 11027316 PMC17331

[B46] Smoller JW Paulus MP Fagerness JA Purcell S Yamaki LH Hirshfeld-Becker D Biederman J Rosenbaum JF Gelernter J Stein MB Influence of RGS2 on anxiety-related temperament, personality, and brain function Arch Gen Psychiatry 2008 65 298 308 10.1001/archgenpsychiatry.2007.48 18316676

[B47] Leygraf A Hohoff C Freitag C Willis-Owen SA Krakowitzky P Fritze J Franke P Bandelow B Fimmers R Flint J Deckert J Rgs 2 gene polymorphisms as modulators of anxiety in humans? J Neural Transm 2006 113 1921 1925 10.1007/s00702-006-0484-8 16736243

[B48] Koenen KC Amstadter AB Ruggiero KJ Acierno R Galea S Kilpatrick DG Gelernter J RGS2 and generalized anxiety disorder in an epidemiologic sample of hurricane-exposed adults Depress Anxiety 2009 26 309 315 10.1002/da.20528 18833580 PMC2666784

[B49] Hettema JM Sun C Chen X Kendler KS Genetic association study between RGS2 and anxiety-related phenotypes Psychiatr Genet 2013 23 92 10.1097/YPG.0b013e32835d70b3 23277133 PMC6935355

[B50] Hettema JM Neale MC Myers JM Prescott CA Kendler KS A population-based twin study of the relationship between neuroticism and internalizing disorders Am J Psychiatry 2006 163 857 864 10.1176/appi.ajp.163.5.857 16648327

[B51] Hettema JM Webb BT Guo AY Zhao Z Maher BS Chen X An SS Sun C Aggen SH Kendler KS Prioritization and association analysis of murine-derived candidate genes in anxiety-spectrum disorders Biol Psychiatry 2011 70 888 896 10.1016/j.biopsych.2011.07.012 21871609 PMC3191234

[B52] Wu Z Puigserver P Andersson U Zhang C Adelmant G Mootha V Troy A Cinti S Lowell B Scarpulla RC Spiegelman BM Mechanisms controlling mitochondrial biogenesis and respiration through the thermogenic coactivator PGC-1 Cell 1999 98 115 124 10.1016/S0092-8674(00)80611-X 10412986

[B53] Lin J Handschin C Spiegelman BM Metabolic control through the PGC-1 family of transcription coactivators Cell Metab 2005 1 361 370 10.1016/j.cmet.2005.05.004 16054085

[B54] Bouayed J Rammal H Soulimani R Oxidative stress and anxiety: relationship and cellular pathways Oxid Med Cell Longev 2009 2 63 67 10.4161/oxim.2.2.7944 20357926 PMC2763246

[B55] Hovatta I Juhila J Donner J Oxidative stress in anxiety and comorbid disorders Neurosci Res 2010 68 261 275 10.1016/j.neures.2010.08.007 20804792

[B56] Parker CC Sokoloff G Cheng R Palmer AA Genome-wide association for fear conditioning in an advanced intercross mouse line Behav Genet 2012 42 437 448 10.1007/s10519-011-9524-8 22237917 PMC3351497

[B57] Nelson EC Agrawal A Pergadia ML Lynskey MT Todorov AA Wang JC Todd RD Martin NG Heath AC Goate AM Association of childhood trauma exposure and GABRA2 polymorphisms with risk of posttraumatic stress disorder in adults Mol Psychiatry 2009 14 234 235 10.1038/mp.2008.81 19229201 PMC3291097

[B58] Liberzon I Taylor SF Phan KL Britton JC Fig LM Bueller JA Koeppe RA Zubieta JK Altered central micro-opioid receptor binding after psychological trauma Biol Psychiatry 2007 61 1030 1038 10.1016/j.biopsych.2006.06.021 16945349

[B59] Fernandes C Paya-Cano JL Sluyter F D’Souza U Plomin R Schalkwyk LC Hippocampal gene expression profiling across eight mouse inbred strains: towards understanding the molecular basis for behaviour Eur J Neurosci 2004 19 2576 2582 10.1111/j.0953-816X.2004.03358.x 15128411

[B60] Hovatta I Tennant RS Helton R Marr RA Singer O Redwine JM Schadt EE Ellison JA Verma IM Lockhart DJ Barlow C Glyoxalase 1 and glutathione reductase regulate anxiety in mice Nature 2005 438 662 666 10.1038/nature04250 16244648

[B61] Letwin NE Kafkafi N Benjamini Y Mayo C Frank BC Luu T Lee NH Elmer GI Combined application of behavior genetics and microarray analysis to identify regional expression themes and gene-behavior associations J Neurosci 2006 26 5277 5287 10.1523/JNEUROSCI.4602-05.2006 16707780 PMC6675305

[B62] Donner J Pirkola S Silander K Kananen L Terwilliger JD Lonnqvist J Peltonen L Hovatta I An association analysis of murine anxiety genes in humans implicates novel candidate genes for anxiety disorders Biol Psychiatry 2008 64 672 680 10.1016/j.biopsych.2008.06.002 18639233 PMC2682432

[B63] Dodman NH Karlsson EK Moon-Fanelli A Galdzicka M Perloski M Shuster L Lindblad-Toh K Ginns EI A canine chromosome 7 locus confers compulsive disorder susceptibility Mol Psychiatry 2010 15 8 10 10.1038/mp.2009.111 20029408

[B64] Williams R Lim JE Harr B Wing C Walters R Distler MG Teschke M Wu C Wiltshire T Su AI A common and unstable copy number variant is associated with differences in Glo1 expression and anxiety-like behavior PLoS One 2009 4 e4649 10.1371/journal.pone.0004649 19266052 PMC2650792

[B65] Distler MG Plant LD Sokoloff G Hawk AJ Aneas I Wuenschell GE Termini J Meredith SC Nobrega MA Palmer AA Glyoxalase 1 increases anxiety by reducing GABAA receptor agonist methylglyoxal J Clin Invest 2012 122 2306 2315 10.1172/JCI61319 22585572 PMC3366407

[B66] Thornalley PJ The glyoxalase system: new developments towards functional characterization of a metabolic pathway fundamental to biological life Biochem J 1990 269 1 11 2198020 10.1042/bj2690001 PMC1131522

[B67] Mannervik B Molecular enzymology of the glyoxalase system Drug Metabol Drug Interact 2008 23 13 27 18533362 10.1515/dmdi.2008.23.1-2.13

[B68] Kromer SA Kessler MS Milfay D Birg IN Bunck M Czibere L Panhuysen M Putz B Deussing JM Holsboer F Identification of glyoxalase-I as a protein marker in a mouse model of extremes in trait anxiety J Neurosci 2005 25 4375 4384 10.1523/JNEUROSCI.0115-05.2005 15858064 PMC6725100

[B69] Szego EM Janaky T Szabo Z Csorba A Kompagne H Muller G Levay G Simor A Juhasz G Kekesi KA A mouse model of anxiety molecularly characterized by altered protein networks in the brain proteome Eur Neuropsychopharmacol 2010 20 96 111 10.1016/j.euroneuro.2009.11.003 20015620

[B70] Distler MG Palmer AA Role of Glyoxalase 1 (Glo1) and methylglyoxal (MG) in behavior: recent advances and mechanistic insights Front Genet 2012 3 250 23181072 10.3389/fgene.2012.00250 PMC3500958

[B71] Fujimoto M Uchida S Watanuki T Wakabayashi Y Otsuki K Matsubara T Suetsugi M Funato H Watanabe Y Reduced expression of glyoxalase-1 mRNA in mood disorder patients Neurosci Lett 2008 438 196 199 10.1016/j.neulet.2008.04.024 18455873

[B72] Eser D Uhr M Leicht G Asmus M Langer A Schule C Baghai TC Mulert C Rupprecht R Glyoxalase-I mRNA expression and CCK-4 induced panic attacks J Psychiatr Res 2011 45 60 63 10.1016/j.jpsychires.2010.05.008 20542521

[B73] Arai M Yuzawa H Nohara I Ohnishi T Obata N Iwayama Y Haga S Toyota T Ujike H Ichikawa T Enhanced carbonyl stress in a subpopulation of schizophrenia Arch Gen Psychiatry 2010 67 589 597 10.1001/archgenpsychiatry.2010.62 20530008

[B74] Politi P Minoretti P Falcone C Martinelli V Emanuele E Association analysis of the functional Ala111Glu polymorphism of the glyoxalase I gene in panic disorder Neurosci Lett 2006 396 163 166 10.1016/j.neulet.2005.11.028 16352396

[B75] Ditzen C Varadarajulu J Czibere L Gonik M Targosz BS Hambsch B Bettecken T Kessler MS Frank E Bunck M Proteomic-based genotyping in a mouse model of trait anxiety exposes disease-relevant pathways Mol Psychiatry 2010 15 702 711 10.1038/mp.2008.146 19139748

[B76] Carboni L Piubelli C Pozzato C Astner H Arban R Righetti PG Hamdan M Domenici E Proteomic analysis of rat hippocampus after repeated psychosocial stress Neuroscience 2006 137 1237 1246 10.1016/j.neuroscience.2005.10.045 16338082

